# 3D Printing in Medical Libraries: A Crash Course in Supporting Innovation in Health Care

**DOI:** 10.5195/jmla.2019.765

**Published:** 2019-10-01

**Authors:** Melanie J. Norton

**Affiliations:** Cushing/Whitney Medical Library, Yale University, New Haven, CT, melanie.norton@yale.edu

Three-dimensional (3D) printing capabilities did not start until the late twentieth century, but some of the ideas behind the process date back to the 1800s. A French “photosculptor” named François Willème demonstrated the world’s first 3D scanning technology in 1859 by using twenty-four cameras to simultaneously photograph subjects from different angles. In 1892, inventor Joseph E. Blanther was awarded a patent for a method of creating 3D topographical maps using a layer method similar to the process used by today’s 3D printers [1].

Fast forward to the twenty-first century, and 3D printers can be found in over 1,000 libraries across the country and even in some private homes. *3D Printing in Medical Libraries: A Crash Course in Supporting Innovation in Health Care* is indeed a crash course on how medical libraries can offer a 3D printing service at their institutions.

In her book, Jennifer Herron shares her firsthand knowledge of establishing a 3D printing lab. The fourteen chapters in Herron’s book are packed with practical information based on her experience at the Indiana University School of Medicine’s Ruth Lilly Medical Library. The chapters are short and succinct and can be read individually; however, it would behoove a librarian who is thinking about establishing a 3D printing lab to read the book from cover to cover.

The benefits of having a 3D printing service in a medical library may seem obvious: 3D-printed models of bones, teeth, and organs are currently being used in anatomy curricula. Providing a 3D printing service can be an opportunity for a medical library to better serve students and enhance scholars’ experiences. Having a 3D printing service also helps solve the problem of cadaver availability, as well as provide a safer alternative to repeated exposure to embalming fluids.

However, there is more to consider than reproducing images into a 3D model. Herron’s chapter on legal concerns of a library providing 3D printing is especially interesting. This chapter deals with intellectual property, copyright, trademarks, and patents. These legalities could be problematic for libraries that provide access to 3D printing equipment. Herron offers practical advice, such as contacting the institution’s office of legal counsel and having a detailed printing policy in place prior to offering such services.

Chapter 8 on data management is another thought-provoking chapter. Data from 3D printing can provide a vast array of information that can enhance productivity and quality. Data such as model weight, print time, infill, print speed, and temperature can be assessed. According to Herron, the data will ultimately provide insights leading to a better print operation. After a library has decided to provide a 3D printing service, “the real fun begins” (p. 93), and Herron offers a practical step-by-step guide to follow in chapter 9.

Throughout the chapters, the author provides definitions of 3D printing terminology and ends the book with a glossary of terms. While terminology in chapters is repeated in the glossary, the explanations of terminology within each chapter provide the reader with an instant definition without having to flip to the back of the book.

Chapter 13 is devoted to opinions from medical librarians who have established 3D printing services. While the chapter is motivating, perhaps these testimonials could have been woven into the chapters themselves. The last chapter provides the 3D researcher with a list of annotated recommended resources and is a librarian’s dream. The resources are arranged by media type including books, journals, magazines, social media, 3D repositories, software, and even conferences and events. Although this book focuses on medical libraries, it is an ideal how-to manual for any librarian who desires to set up a 3D printing service but does not know where to start.

***Melanie J. Norton, MLIS****, melanie.norton@yale.edu, Cushing/Whitney Medical Library, Yale University, New Haven, CT*
